# Nanotopological plate stimulates osteogenic differentiation through TAZ activation

**DOI:** 10.1038/s41598-017-03815-5

**Published:** 2017-06-15

**Authors:** Jun-Ha Hwang, Dong-Hyun Lee, Mi Ran Byun, A. Rum Kim, Kyung Min Kim, Jung Il Park, Ho Taek Oh, Eun Sook Hwang, Kyu Back Lee, Jeong-Ho Hong

**Affiliations:** 1 Department of Life Sciences, School of Life Sciences and Biotechnology, Korea University, Seoul, 02841 Korea; 2 Department of Interdisciplinary Bio/Micro System Technology, College of Engineering, Korea University, Seoul, 02841 Korea; 3 College of Pharmacy and Graduate School of Pharmaceutical Sciences, Ewha Womans University, Seoul, 03760 Korea; 4 School of Biomedical Engineering, College of Health Science, Korea University, Seoul, 02841 Korea

## Abstract

The topographical environment, which mimics the stem cell niche, provides mechanical cues to regulate the differentiation of mesenchymal stem cells (MSC). Diverse topographical variations have been engineered to investigate cellular responses; however, the types of mechanical parameters that affect cells, and their underlying mechanisms remain largely unknown. In this study, we screened nanotopological pillars with size gradient to activate transcriptional coactivator with PDZ binding motif (TAZ), which stimulates osteogenesis of MSC. We observed that a nanotopological plate, 70 nm in diameter, significantly induces osteogenic differentiation with the activation of TAZ. TAZ activation via the nanotopological plate was mediated by actin polymerization and Rho signaling, as evidenced by the cytosolic localization of TAZ under F-actin or Rho kinase inhibitor. The FAK and MAPK pathways also play a role in TAZ activation by the nanotopological plate because the inhibitor of ERK and JNK blocked nanopattern plate induced osteogenic differentiation. Taken together, these results indicate that nanotopology regulates cell differentiation through TAZ activation.

## Introduction

Tissue engineering is generally applied for reconstruction of human tissue by converging methods, such as material science and life science. Various types of artificial insertions such as ceramics and biocompatible metals have been generated and examined in the human body directly. Accordingly, several studies have investigated methods of overcoming the associated inflammation and functional failure^1^. However, stem cells immerged as an influential option for tissue engineering through noticeable *in vitro* studies, and mesenchymal stem cells (MSC) have been commonly used for this purpose. Owing to their multipotency and immune-modulatory characteristics, MSC are applied in numerous medical areas such as tissue repair and cell therapy^2–5^. Among the many attributes of MSC, their capacity for osteogenesis, a multipotent differential orientation, is an important mechanism for bone formation and regeneration in tissue engineering.

The parameters of the *in vitro* microenvironment for regulating MSC differentiation, which promotes differentiation to osteoblastic lineages, have been investigated in tissue engineering and regenerative medicine. Among various parameters related to the *in vitro* microenvironment, soluble chemical factors have typically been studied, and it has been shown that the expression of several specific proteins affects the differentiation status of stem cells^6, 7^. Recently, it was demonstrated that physical/mechanical factors of extracellular environments, as well as soluble chemical factors, can induce various cellular responses such as differentiation, self-renewal, and migration. The physical/mechanical factors include the hardness of materials and size, as well as pitch and shape of micro/nanostructures on the cultural plates^8–10^.

Each factor was relevant for cell/plate interaction. Specifically, the micro/nano-structured plate revealed intriguing results for cellular response, which is related to cell plate adhesion interaction. Those studies indicated that periodic nanosize-fabricated structure induces differentiation of MSC and the pitch of the nanostructure triggers cells to induce cellular migration, differentiation, or maintenance in an undifferentiated state^11–15^. In relation with this, it is notable that several types of extracellular matrix have mesh-like nanoscale structures, with fiber diameter and pore size^16^. It is shown that cells sense nanoscale differences in the distribution of cell adhesion ligands in response to artificial substrates^17–19^.

For understanding of the relationship between cell response and topographical features, selective diversification of topological parameters such as diameters, pitch has to be carried out. Anodic aluminum oxide (AAO) has high-quality regularity of hexagonal nanopore array and is simple to produce. Furthermore, it provides suitable templates for simple and fast nanofabrication. AAO also has a chemical property that etches chemically with acid-based solutions, which means that the time-controlled etch process produces AAO with different hole sizes on a single plate^20, 21^. The nanopattern with size-gradient, fabricated via a time controlled etch process, provides suitable factors to study topology mediated cell response.

Transcriptional co-activators with PDZ-binding motif (TAZ)/Yes-associated protein (YAP) are known to regulate cell proliferation, differentiation, and stemness maintenance through diverse signaling pathways such as Wnt, GPCR, and Hippo^22–24^. Upon activation, TAZ/YAP functions as a transcriptional modulator for target gene expression. For regulation of cellular differentiation, TAZ promotes osteogenesis but represses adipogenesis^24^. TAZ physically interacts with Runx2, an osteogenic transcription factor, and stimulates Runx2 target genes. However, TAZ inhibits PPAR-gamma-mediated transcription to suppress adipogenesis. Recent studies demonstrate that TAZ is also regulated by mechanical forces induced by the physical properties of the extracellular matrix (ECM). Specifically, stiffness of the ECM molecule or shear stress induced by extracellular fluid regulates intracellular actin dynamics, which further regulates the stability of the TAZ protein^25, 26^.

Since the topology of the ECM regulates cellular proliferation and differentiation, we screened a nanotopological plate to determine the optimal size for TAZ activation following osteogenic induction. We identified the optimal size of nanotopological pillars for osteogenic differentiation, and demonstrated that the Rho and MAPK pathways act as mediators for nanotopology-induced TAZ activation, which regulates differentiation of human MSC.

## Results

### An oxalic gradient nanotopological plate of 70 nm diameter is optimal for TAZ activation

**Figure 1:**
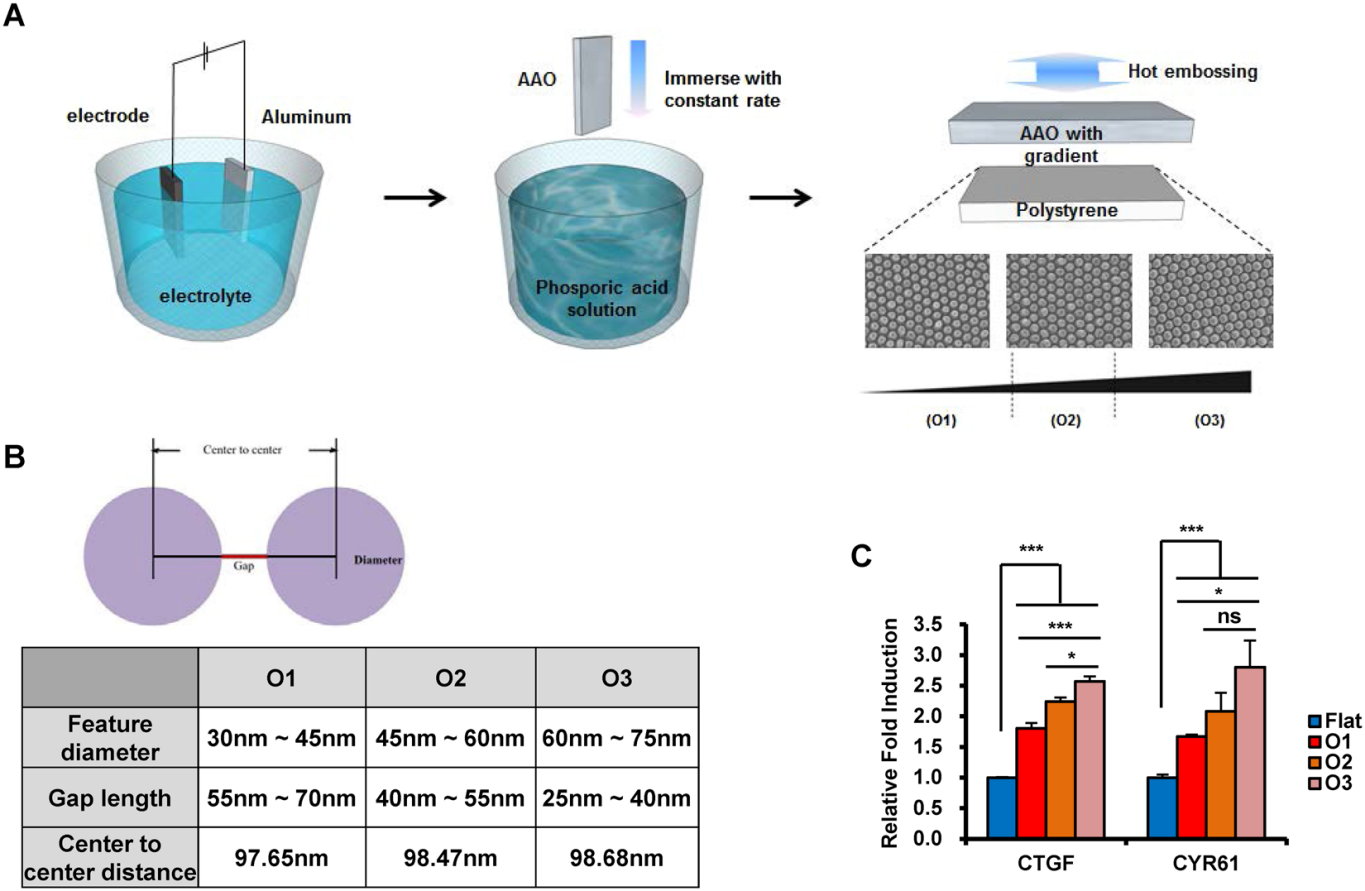
Preparation of nanopattern plates and screening of nanopattern surfaces with size gradients for TAZ target-gene induction. (**A**) An electrochemically polished aluminum foil was prepared in oxalic acidic condition. The anodizing process for fabrication of anodic aluminum oxide (AAO) was performed and the pore size gradient on AAO was produced using a tensiometer. Polystyrene plates with gradient nanopillar patterns were fabricated via the thermal imprint process. (**B**) Feature diameter, gap length, and center-to-center distance of O1, O2, and O3 nanopattern surfaces manufactured in the panel (**A**) are listed. (**C**) MSC were seeded on flat or nanopattern plates (O1, O2, and O3) and harvested after 48 hr. Transcript levels of CTGF and CYR61 were analyzed by quantitative real-time PCR. Asterisks mean statistical significance. n = 3, *p < 0.05, ***p < 0.005, ns = not significant, t-test.

Nanostructures influence the differentiation and maintenance of undifferentiated stem cells^27^. However, it is largely unknown how stem cell differentiation is regulated on nanostructures of any size, and the mechanism that induces this phenomenon. To determine the optimal size of nanostructure, nanotopology with various gradient sections was fabricated (Fig. 1A and B). Initially, the O1 (30–45 nm diameter), O2 (45–60 nm diameter), and O3 sections (60–75 nm diameter) of gradient nanotopological plates were created with different etching times (Fig. 1A and B). To screen for the specific range of feature size optimized, we investigated the activation of TAZ, since TAZ is a transcriptional regulator of cellular proliferation and differentiation^25, 26, 28, 29^. Human MSC were plated on gradient nanotopological plates and the transcription of TAZ target genes including connective tissue growth factor (CTGF) and cysteine-rich angiogenic inducer 61 (CYR61) were analyzed by quantitative RT-PCR (qRT-PCR). As shown in Fig. 1C, increased target gene transcription was observed in all nanotopoloical plates. Among them, the O3 section showed the highest transcription of *CTGF* and *CYR61* genes.

**Figure 2:**
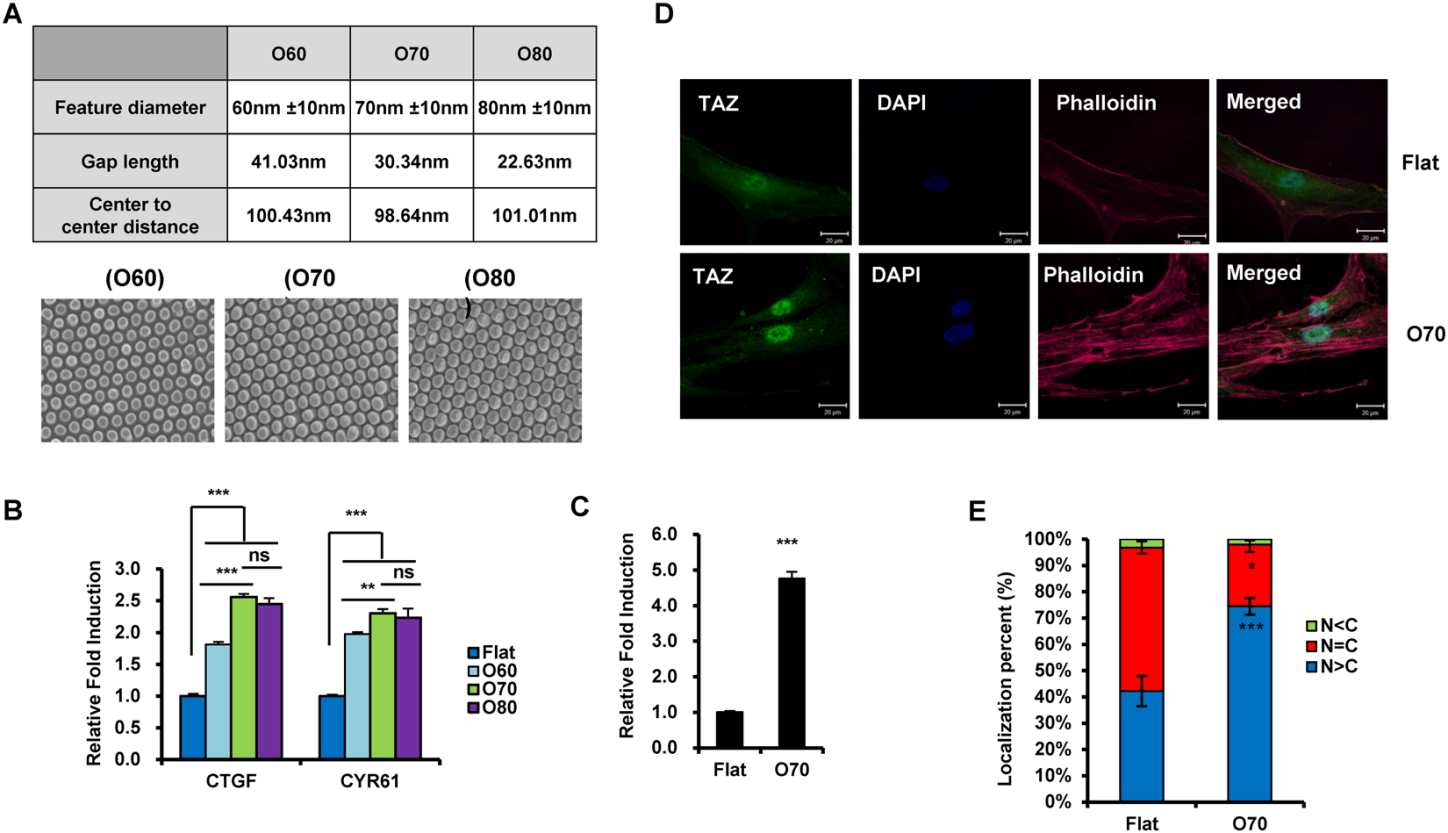
The O70 nanopattern plate is optimal for TAZ nuclear localization and activation. (**A**) Features of the O60, O70, and O80 nanopattern plates. Feature diameter, gap length between features, and the distance of the center-to-center were listed, and the SEM image of each nanopattern plate is shown. (**B**) MSC were seeded on flat or nanopattern plates and harvested after 48 hr. Transcript levels of CTGF and CYR61 were analyzed by quantitative real-time PCR. Asterisks mean statistical significance. n = 3, **p < 0.01, ***p < 0.005, ns = not significant, t-test. (**C**) MSC were transfected with CTGF-luc. After 16 hr, cells were re-seeded on flat or nanopattern plates, and luciferase activity was measured using a luciferase reporter gene assay system 24 hr after re-seeding. n = 3, ***p < 0.005, t-test. (**D**) MSC were seeded on flat or nanopattern plates and immunostained with TAZ specific antibody 24 hr after seeding. F-actin and the cell nucleus were counterstained with phalloidin and DAPI, respectively. (**E**) Immunostained cells in panel (**D**) were counted using the image J software, and the cell population for nuclear dominant, cytosol dominant, and evenly distributed TAZ was calculated. n = 3, *p < 0.05, ***p < 0.005, t-test.

To further define the optimal size of nanopatterns, the O3 section was further divided into fixed nanopatterns size. O60 (number indicates mean diameter of nanostructure), O70, and O80 nanopatterns were produced to analyze TAZ activation, since the O3 section has 60–75 nm of feature diameters (Fig. 2A). Among the three nanotopological patterns, O70 had the highest transcriptional activity compared to the others (Fig. 2B). Therefore, the results suggest that nanopatterns of 70 nm diameter is optimal for TAZ activation. For further investigation of transcriptional activation of TAZ on the O70 plate, we introduced the CTGF promoter containing the luciferase reporter plasmid (CTGF-luc), which can be stimulated by TAZ, into human MSC. Cells were seeded on flat and O70 plates. As shown in Fig. 2C, luciferase activity increased dramatically in O70 plates relative to flat plates. These data also suggest that nanotopological plates activate TAZ transcriptional activity. Next, in order to further study TAZ activation, we assessed the cellular location of TAZ using immunocytochemistry. TAZ is a transcriptional regulator, which should be located in the nucleus for its function^30^. The cellular location of TAZ on flat or nanotopological plates was visualized by fluorescence. As shown in Fig. 2D and E, increased nuclear localization was observed in the O70 plate. Therefore, the results indicate that nanotopological signals activate TAZ.

### Nanotopological plate activates osteogenic differentiation

**Figure 3:**
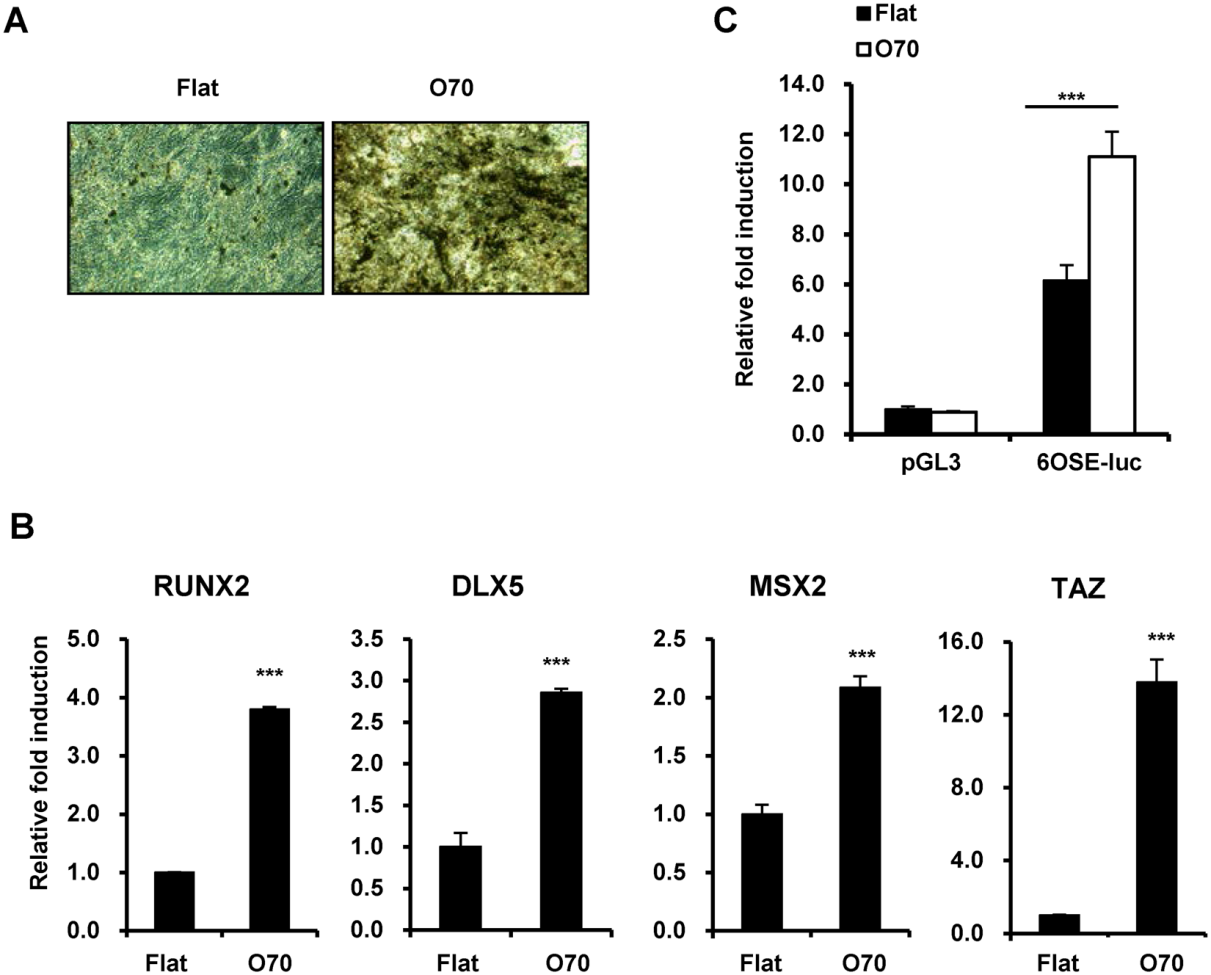
The O70 nanopattern plate promotes osteogenesis of MSC. (**A**) MSC on flat or nanopattern plates were differentiated for 14 days and calcium deposition was analyzed by Von Kossa staining. (**B**) Cells in (**A**) were harvested and transcripts of *RUNX2*, *DLX5*, *MSX2*, and *TAZ* were analyzed by qRT-PCR. n = 3, ***p < 0.005, t-test. (**C**) MSC were transfected with pGL3 basic or 6OSE2-luc plasmid along with RUNX2 expression plasmid and re-seeded on flat or nanopattern plates after 16 hr. At 24 hr after re-seeding, cells were harvested, and luciferase activity was analyzed. n = 3, ***p < 0.005, t-test.

MSC have multipotency potential to differentiate into a variety of cell types, including osteoblasts. TAZ is known to function as a transcriptional modulator that regulates osteogenesis^24^. To investigate whether nanotopological plate driven TAZ activation regulates MSC differentiation, we evaluated the osteogenic potential of human MSC on normal flat and O70 plates. As shown in Fig. 3A, osteogenesis was upregulated in the O70 plate, as evidenced by increased mineralization through Von Kossa staining. The expression of osteoblast marker genes including *RUNX2*, *DLX5*, *MSX2*, and *TAZ* also increased in the O70 plate (Fig. 3B). Next, to investigate whether the upregulated osteogenic marker genes are driven by RUNX2, we introduced a RUNX2 binding site containing a luciferase reporter plasmid (6OSE2-luc), along with a RUNX2 expression vector into human MSC. The cells were seeded on control flat and O70 plates. On the O70 plate, we observed an approximately two-fold increase in luciferase activity (Fig. 3C). Therefore, the results indicate that the O70 plate stimulates osteogenic differentiation through RUNX2-mediated gene transcription.

### TAZ is required for the regulation of MSC differentiation by nanotopological plate

**Figure 4:**
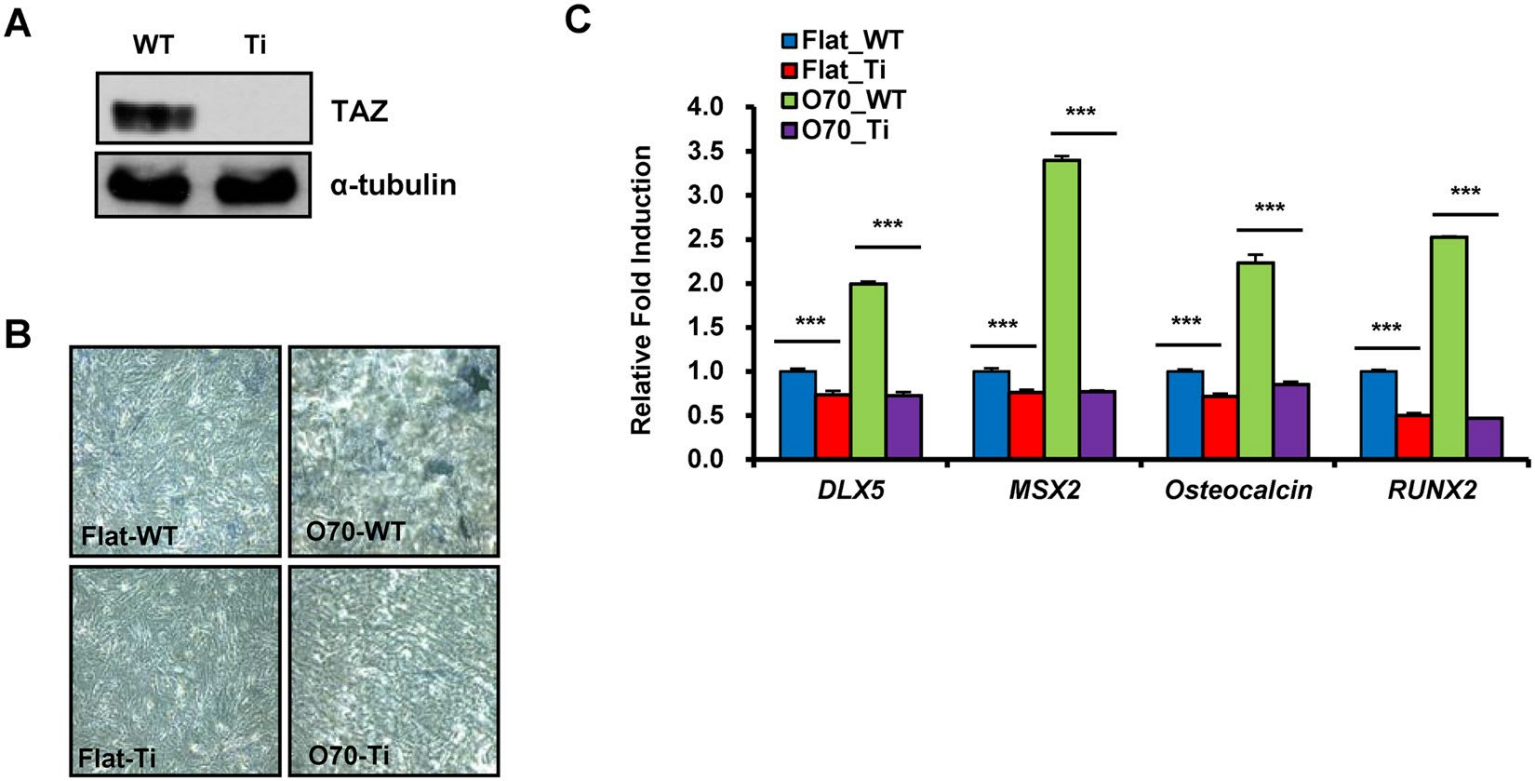
TAZ is required for the osteogenic differentiation of MSC by nanopattern plates. (**A**) TAZ knockdown human MSC were generated using lentivirus-mediated transduction of shRNA. The cells were analyzed by immunoblotting to prove TAZ knockdown. Full length blots cropped for representative Figure are shown in Supplementary Figure S3. (**B**) Wild type and TAZ knockdown MSC were seeded on flat or nanopattern plate and differentiated to osteoblasts. Six days after differentiation, cells were stained using the alkaline phosphatase staining method. (**C**) Cells in panel (B) were harvested and the transcripts of osteogenic marker genes were analyzed by qRT-PCR. Data are shown as relative fold induction. n = 3, ***p < 0.005, t-test.

To investigate further the requirement of TAZ for nanotopological regulation of MSC differentiation, we generated TAZ knockdown human MSC, using TAZ shRNA-producing lentivirus. TAZ knockdown was confirmed by western blot analysis (Fig. 4A). Next, wild type and TAZ knockdown cells were plated on flat or O70 plates to induce differentiation. As shown in Fig. 4B, cells on the O70 plate showed significantly induced alkaline phosphatase activity compared to cells on the flat plate. However, the induced alkaline phosphatase activity decreased in TAZ knockdown cells, indicating that the increased osteogenic potential on the O70 plate is regulated by TAZ. Indeed, the expression of osteogenic marker genes including *DLX5*, *MSX2*, *Osteocalcin*, and *RUNX2* was induced in wild type cells, but not in TAZ knockdown cells on the O70 plate (Fig. 4C). This indicates that TAZ is an important regulator of osteogenic differentiation induced by nanotopological plates.

### Nanotopological plate induced actin polymerization is important for TAZ activation

**Figure 5:**
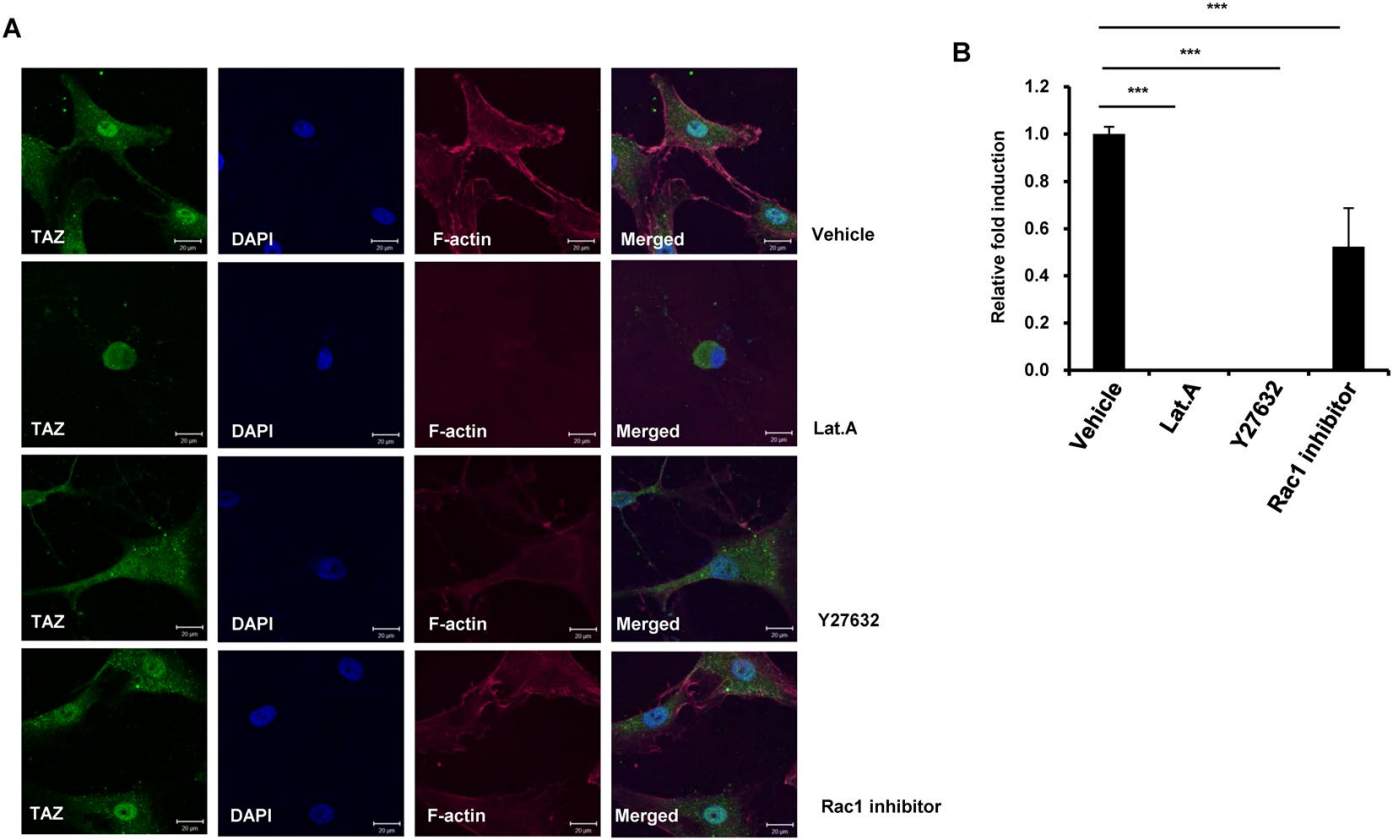
Inhibition of Rho/ROCK/F-actin represses TAZ nuclear localization and TAZ target-gene expression induced by nanopattern plates. (**A**) MSC were seeded on O70 nanopattern plates and 24 hr after seeding, latrunculin A, Y27632, and Rac1 inhibitors were added for 4 hr. Cells were stained with TAZ specific antibody and counterstained with phalloidin and DAPI for visualization of filamentous actin and nucleus, respectively. (**B**) Cells in panel (**A**) were harvested and total RNA was prepared. TAZ target gene CTGF was analyzed by qRT-PCR. n = 3, ***p < 0.005, t-test.

The activation of TAZ by mechanical input is regulated by actin polymerization of the cytoskeleton, as evidenced by cytosolic retention of TAZ and the repression of TAZ target genes by an actin polymerization inhibitor^25^. We assessed whether nanotopology induced nuclear localization of TAZ and whether its transcriptional activity is regulated by actin polymerization. As shown in Fig. 5A, MSC on the O70 plate showed significant nuclear localization of TAZ, and had a polymerized actin cytoskeleton (Fig. 5A). However, in the presence of latrunculin A, an actin polymerization inhibitor, cells were shrunk and rounded, and TAZ was predominantly located in the cytosol. These results indicate that TAZ nuclear localization on the O70 plate is induced by polymerization of the filamentous actin structure. Next, to study whether cell spreading and actin polymerization are driven by Rho GTPase family proteins, cells on the O70 plate were treated with Rho signaling inhibitor, Y27632, and Rac1 inhibitors. As shown in Fig. 5A, Y27632 significantly inhibited nuclear localization of TAZ and F-actin formation. However, the Rac1 inhibitor did not have any significant effects. Thus, the results suggest that Rho GTPase plays an important role in nanotopology induced signaling. Next, to further investigate the effect of the inhibitors in gene transcription, CTGF expression was analyzed by qRT-PCR. As shown in Fig. 5B, Latrunculin A and Y27632 dramatically suppressed *CTGF* gene expression, whereas the Rac1 inhibitor did not. This indicates that TAZ nuclear localization and activation by nanotopological plate is mediated by actin polymerization and Rho signaling pathway.

### TAZ activation by nanotopological plate is regulated by the FAK and MAPK pathways

**Figure 6:**
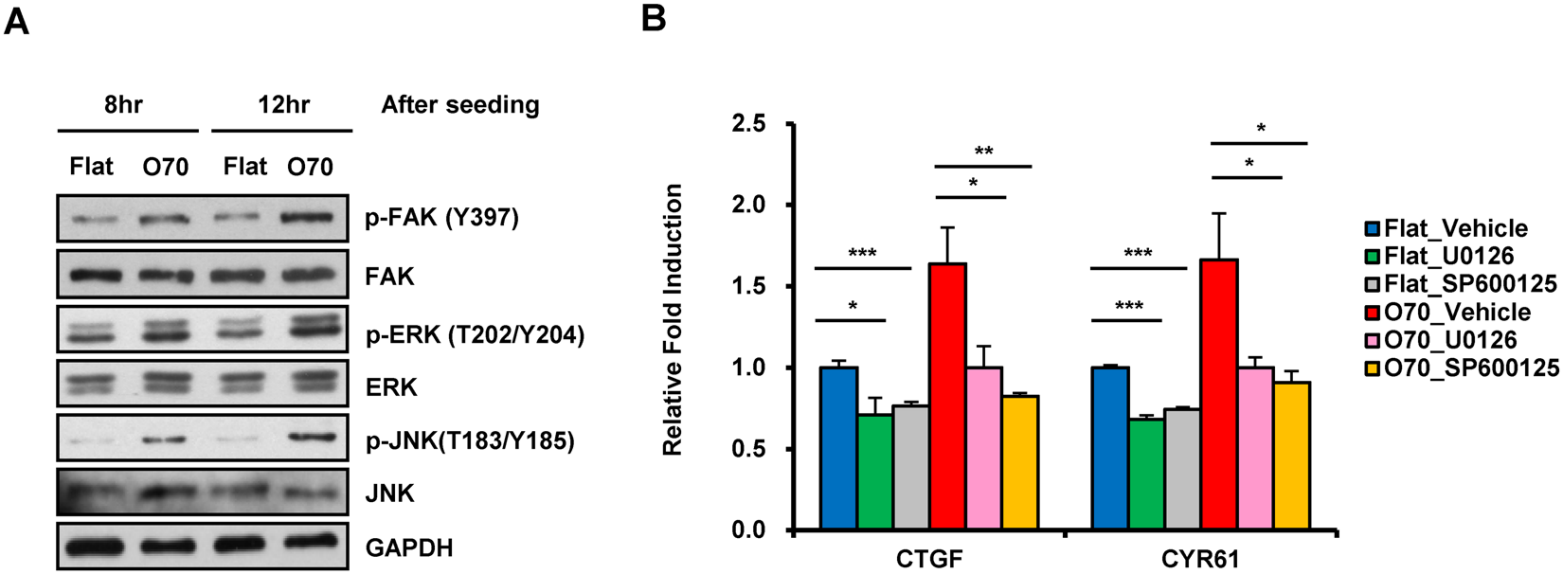
ERK/JNK is activated by nanopattern plate and involved in TAZ target-gene expression induced by nanopattern plates. (**A**) MSC were seeded on flat or O70 nanopattern plates and harvested 8 hr and 12 hr after seeding. Then p-FAK, FAK, p-ERK, ERK, p-JNK, and JNK were analyzed by western blot. GAPDH was used as loading control. Full length blots cropped for representative Figure are shown in Supplementary Figure S3. (B) MSC seeded on flat or O70 nanopattern plates were treated with vehicle, 10 μM U0126, and 10 μM SP600125 for 12 hr. Cells were then harvested, and CTGF and CYR61 expression was analyzed by qRT-PCR with GAPDH as a reference. Data was shown as relative fold induction. n = 3, *p < 0.05, **p < 0.01,***p < 0.005, t-test.

It was shown that the nanotopological pattern regulates the differentiation of primary human osteoblasts by modulating integrin clustering and focal adhesion formation with activation of FAK^31, 32^. In addition, MAPK signaling is important for osteogenic differentiation^33, 34^, and functions as a downstream component of FAK signaling^35^. Notably, we observed that TAZ acts as a mediator of the MAPK pathway to control cellular proliferation and differentiation in response to soluble growth factor or mechanical stress^26, 36^. Therefore, we analyzed the involvement of FAK and MAPK pathways in O70 plate induced TAZ activation. At 8 and 12 hrs after cell plating on flat and O70 plates, cell lysates were collected and the activity of FAK, ERK, and JNK was analyzed using phospho-specific antibodies. As shown in Fig. 6A, increased phosphorylation of FAK, ERK, and JNK was observed in O70 plates, suggesting that nanotopological pattern stimulates these key signaling proteins. The results were further verified by using the ERK or JNK inhibitors, U0126 or SP600125, respectively. The inhibitor treatment suppressed nanotopology-induced CTGF and CYR61 induction (Fig. 6B). Therefore, the results indicate that the FAK-MAPK pathway is involved in nanotopology-induced TAZ target gene expression.

## Discussion

Various physical properties such as shear stress, stiffness of organ surface, and specific surface features comprise the upstream cues of mechanotransduction, which induce intracellular signaling cascades. Many nanotopological plate features exist in the ECM, which contains mixture of ridges, fibers, pores, pits, and grooves. With chemical signaling factors, these nanotopological features induce highly complex signaling networks in living organisms and impact on their biological function and behavior^37–39^. It was reported that the nanotopological plate stimulates osteogenesis through RUNX2 target gene activation, but the mechanism was not clearly understood^27, 40, 41^. In our study, we developed a nanotopological plate with size gradient and observed that a nanotopological pillar of 70 nm diameter is optimal for osteogenic differentiation. The nanopattern stimulates the transcriptional co-regulator, TAZ and facilitates TAZ mediated osteogenic marker gene expression, marking TAZ as a mediator for nanotopolgy driven cellular signaling. Indeed, we observed that TAZ activation was induced by Rho and MAPK signaling pathways (Figs 5 and 6). This study provides a novel link between nanotopology and the cellular signaling pathways that impact cellular function.

Fiedler *et al*. investigated the osteogenic potential of nanopillar arrays with diameters (10–30 nm), inter-pillar distances (50–120 nm), and heights (20–50 nm), showing that higher nanopillars have better osteogenic potential than lower nanopillars^42^. Notably, they also observed that increased diameter is associated with increased osteogenic potential in nanopillars 50 nm in height. In our study, we observed that increased diameters up to 80 nm enhanced the potential for osteogenesis compared to smaller diameters. Similar observations have also been reported, although other topological patterns were analyzed in those studies. Nanotubes of 70–100 nm in diameter increases stem cell elongation and osteogenic differentiation compares to small nanotubes of 30 nm in diameter^43^. It was also shown that TiO_2_ nanotubes of 70 nm in diameter were the optimal dimension for the osteogenic differentiation of human adipose-derived stem cells. They were also shown to stimulate methylation of histone H3 at lysine 4 (H3K4) in the promoter of Runx2 and osteocalcin^44^. These results indicate that there is an optimal nanotopological diameter, which facilitates transcriptional activation for osteogenic differentiation. While, it is known that attached cell area is an important factor for osteogenic process, but we did not observe a significant difference between flat and O70 nanotopological surface (Supplementary Fig. S1).

In addition to nanotopological parameter, other biocompatible parameters including hardness and elasticity are also important for optimal osteogenic differentiation. In relation with this, Sommer *et al*. addressed key determinants of biocompatibility called as biomimetic triangle^45^. The report suggest that the parameters of biomimetic triangle should be considered for generation of optimal osteogenic plates.

Recently, stem cell biology has received a lot of attention because of its potential in clinical applications. MSC is used as a source for the treatment of diseases including myocardial infarction and spinal cord injury. Accordingly, there are several MSC-based therapies in clinical development^5^. However, it is known that obtaining sufficient amounts of MSC for cell therapy is difficult due to senescence after multiple passages. From this point of view, a nanotopological plate could be a useful tool for cell amplification. We observed that cellular proliferation markers such as CTGF and CYR61 are significantly amplified on an O70 plate (Fig. 2B). We also observed that that O70 plate stimulates cell growth compare to flat plate by analyzing cell proliferation assay (Supplementary Fig. S2). This provides better conditions for MSC amplification compared to that by the use of a flat plate. It would be interesting to study whether the O70 nanotopological plate delays entry into senescence.

**Figure 7:**
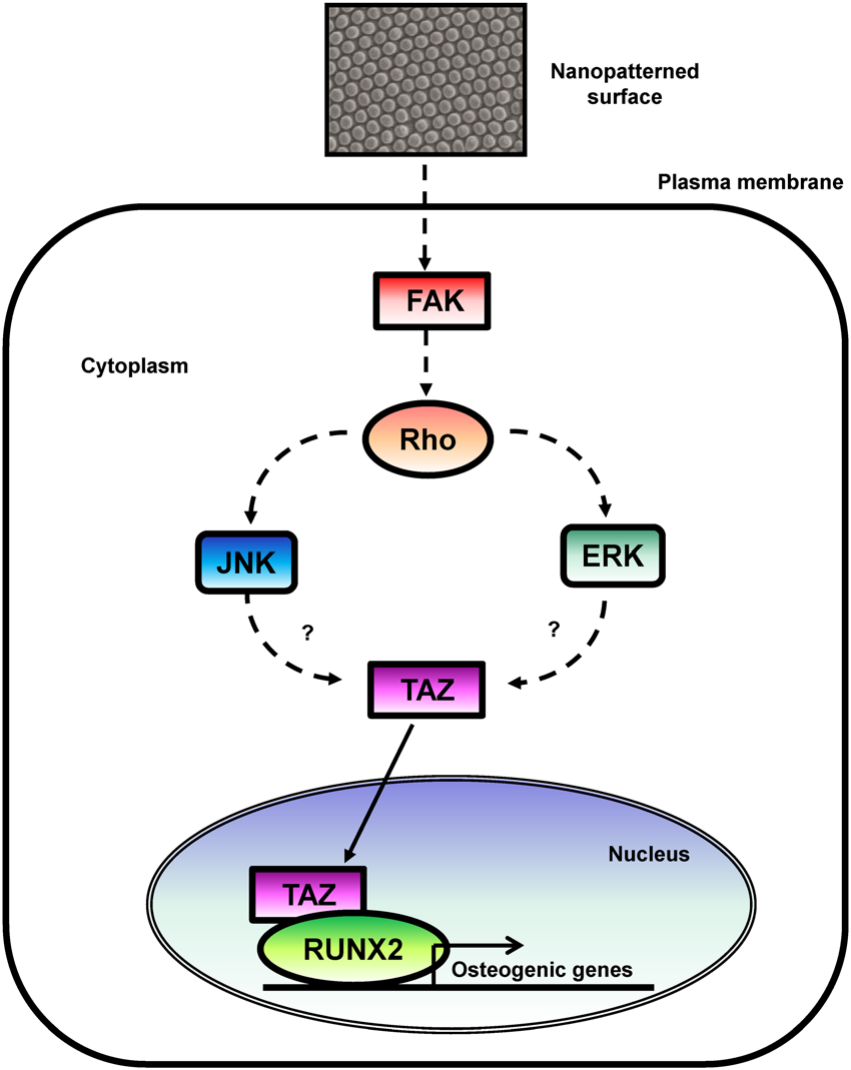
Experimental model. Nanotopological signal stimulates FAK and following downstream ERK and JNK, which activates TAZ and facilitates its nuclear localization. Activated TAZ stimulates RUNX2-mediated gene transcription and induces osteogenic differentiation.

In this study, we observed that TAZ mediates cellular signaling upon nanotopology. Rho signaling and F-actin are upstream signaling components in this process, and the MAPK pathway is also involved (Fig. 7). Indeed, we observed increased TAZ nuclear localization in C3H10T1/2 cells, which overexpress constitutively active ROCK (unpublished observation), indicating that TAZ is a downstream effector of Rho signaling. In addition, integrins are cellular sensors for ECM and activate FAK, which regulates Rho signaling components^46^. Therefore, there appears to be a close connection between nanotopolgy-induced TAZ activation and the integrin-FAK-ROCK pathway. Additional detailed mechanistic studies should be conducted to determine the overall signaling networks that regulate TAZ activity by nanotopography. In conclusion, we identified the optimal size of nanotopological patterns for the activation of TAZ, which induces osteogenic differentiation of MSC through the activation of FAK and MAPK.

## Methods

### Preparation of the nanopatterned plate

Aluminum foil (99.999%, Goodfellow, UK) was electrochemically polished with a perchloric acid/ethanol mixture solution (4:1 v/v, Samchun, KR) at 20 V and 7 °C. An anodizing process to fabricate highly ordered porous anodic aluminum oxide (AAO) was performed in two steps, using oxalic acid solution (0.3 M oxalic acid in 1 L DI water, Sanchun, KR). Mirror-polished aluminum foil was anodized at 40 V and 15 °C for 15 hr with oxalic acid solution and etched out with chromic acid solution (9 g chromic acid & 20.3 mL phosphoric acid in 500 mL DI water, Samchun, KR). A second anodization was performed with the previously used oxalic acid solution for 3 min at 40 V and 15 °C. The fabricated AAO was gradually immersed in phosphoric acid solution at a constant dipping rate (5.765 g in 500 mL DI water) using a tensiometer to produce a pore size gradient on AAO. The AAO gradient plate was hydroxylated using piranha solution (35% H_2_SO_4_:H_2_O_2_ ¼ 7:3, v/v, Samchun, KR), and the heptadecafluoro-1,1,2,2-tetrahydrodecyl-trichlorosilane (HDFS, Sigma-Aldrich, US) monolayer was coated via a self-assembled reaction on the AAO plate, using a 3-mM HDFS solution of *n*-hexane.

Polystyrene plates with gradient nanopillar patterns, fabricated by a thermal imprint process using a nanoimprint device (NX-2000, Nanonex, US), were used for cell culture. Biocompatible polystyrene plates (1 T, Goodfellow, UK) were prepared for imprint, heated up to 130 °C for 20 min, and then pressured at 100 psi for 90 sec at 165 °C. After pressing, the sample was allowed to cool down to 40 °C and AAO was detached from the polystyrene substrate. A sterilization step was required for the polystyrene nanopattern prior to cell cultivation. Ethanol solution (70% v/v) was used for soaking for 1 hr. The plate was then thoroughly dried and sanitized with plasma sterilizer for 1 hr.

### Cell culture

Human MSC were purchased from Lonza and maintained in DMEM containing 10% fetal bovine serum (FBS) (Hyclone, US) at 37 °C and 5% CO_2_ atmosphere. The growth media was renewed every 2 days during culture.

### Antibodies

FAK (#3285), p-FAK (#3283), ERK (#9102), p-ERK (#9101), JNK (#9252), and p-JNK (#9251) antibodies were purchased from Cell Signaling Technology (US). The TAZ (560235) antibody was purchased from BD Pharmigen (US). GAPDH (sc-32233) and the α-tubulin (sc-5286) antibody was from Santa Cruz Biotechnology (US).

### Osteogenic differentiation

For osteogenesis, hMSC were plated on flat or nanopatterned plates at a density of 2 × 10^4^/cm^2^. After 24 hr, osteogenesis was triggered by changing the media to new media containing stimulants for osteogenic differentiation (10% FBS containing DMEM supplemented with 50 μg/ml ascorbic acid, 0.1 μM dexamethasone, and 10 mM β-glycerophosphate, Sigma-Aldrich, US). The differentiation media was changed every 2 days.

### Alkaline phosphatase staining

Differentiated osteoblasts were fixed with 3.7% formaldehyde solution and incubated with staining solution (0.1 mg/ml of naphthol AS-MX phosphate, 0.5% *N*,*N*-dimethylformamide, 2 mM MgCl_2_, and 0.6 mg/ml Fast Blue BB salt, Sigma-Aldrich, US) for 30 min. Then, stained cells were washed with dH_2_O and images were captured under a microscope connected to digital camera (Olympus, JP).

### Von Kossa staining

Differentiated cells were rinsed with PBS and fixed with 3.7% formaldehyde for 10 min. The cells were then washed with dH_2_O, and incubated in 2% silver nitrate (Sigma-Aldrich, US) under UV exposure for 30 min. Next, the cells were washed with dH_2_O and incubated in 0.3% sodium thiosulfate (Sigma-Aldrich, US) for 5 min. Finally, cells were washed again with dH_2_O and air-dried.

### Western blotting

Cells were harvested with 1X SDS sample buffer and briefly sonicated. The samples were then boiled at 95 °C for 5 min. Total cell lysates were separated by SDS-PAGE and blotted on to a PVDF membrane (Merck, DE). The membrane was then blocked with 5% non-fat dry milk in TBST for 1 hr. Next, the membrane was incubated with primary antibody diluted in TBST with 5% BSA (Bovogen, AU) at 4 °C overnight. The membrane was then washed three times with TBST for 5 min. Then, secondary antibody (Enzo Life Sciences, US) diluted in TBST with 5% non-fat dry milk was added to the membrane, which was then incubated at room temperature for 1 hr. The desired protein band was detected using the ECL system (Absignal, Abclon, KR).

### Quantitative real-time PCR for gene expression analysis

Total RNA was prepared using TRIzol reagent (Invitrogen, US) and chloroform. cDNA was synthesized using M-MLV reverse transcriptase (Thermo, US). cDNA samples were then analyzed by quantitative real-time PCR (LightCycler480, Roche, CH). The sequences of the primers used are shown in Table 1.

**Table 1:** Primers for qRT-PCR.

Gene	Direction	Sequence
*CTGF*	Forward	5′-CGACTGGAAGACACGTTTGG-3′
	Reverse	5′-CAGGTCTTGGAACAGGCG-3′
*CYR61*	Forward	5′-CAGGTCTTGGAACAGGCG-3′
	Reverse	5′-GGTTGTATAGGATGCGAGGCT-3′
*DLX5*	Forward	5′-CTACAACCGCGTCCCAAG-3′
	Reverse	5′-GCCATTCACCATTCTCACCT-3′
*MSX2*	Forward	5′-CTACCCGTTCCATAGACCTGT-3′
	Reverse	5′-GAGAGGGAGAGGAAACCCTTT-3′
*Osteocalcin*	Forward	5′-TGAGAGCCCTCACACTCCTC-3′
	Reverse	5′-ACCTTTGCTGGACTCTGCAC-3′
*RUNX2*	Forward	5′-AGAGGTACCAGATGGGACTGT-3′
	Reverse	5′-GGTAGCTACTTGGGGAGGATT-3′
*GAPDH*	Forward	5′-ACATCGCTCAGACACCATG-3′
	Reverse	5′-TGTAGTTGAGGTCAATGAAGGG-3′

### Immunocytochemistry

hMSC were seeded on flat or nanopatterned plates at a density of 1 × 10^4^/cm^2^. Cells were fixed with 2% formaldehyde for 15 min and permeabilized with 0.3% Triton X-100. After blocking with 5% normal goat serum (Vector laboratories, US) for 1 hr, cells were incubated with primary antibody diluted in 1% BSA at 4 °C overnight. After three washes with PBS, cells were incubated with fluorochrome-conjugated secondary antibody (Invitrogen, US) diluted in 1% BSA at room temperature for 2 hr. Cells were then washed again with PBS three times and counterstained with phalloidin (Invitrogen, US) for 20 min. Cells were rinsed with PBS, counterstained with DAPI (Vector laboratories, US), and then mounted. Images were obtained by confocal microscopy (Carl Zeiss, LSM510 META, DE). For cell counting, the Image J software was used.

### Luciferase reporter gene assay

hMSC were transfected with CTGF-luc or 6OSE2-luc luciferase reporter construct, using the Xtremegene HP transfection reagent (Roche, CH). In the case of the 6OSE2-luc gene assay, a myc-tagged Runx2 expression plasmid was co-tranfected. A renilla luciferase reporter construct was co-transfected as an internal reference in all cases. After 16 hr of transfection, cells were re-seeded on flat or nanopatterned plates. Cells were incubated for 24 hr and lysed using 1X passive lysis buffer (Promega, US). The luciferase activity of cell lysates was analyzed using the Dual Luciferase^®^ Reporter Assay System (Promega, US) and a luminometer (GLOMAX, Promega, US). All experiments were performed in triplicate.

### Statistical analysis

Data are presented as means ± SE. To confirm the significance of differences between experimental groups, statistical analysis for experimental results was performed by Student’s *t*-test. (*p < 0.05, **p < 0.01, ***p < 0.005).

## Electronic supplementary material

Supplementary Information
