# Supplementary material for: Nanotopological plate stimulates osteogenic differentiation through TAZ activation
Source: Sci Rep. 2017 Jun 15;7:3632. doi: 10.1038/s41598-017-03815-5 (PMC5472602; doi:10.1038/s41598-017-03815-5)
Supplement: Supplementary file 1 — Supplementary Information [file 41598_2017_3815_MOESM1_ESM.pdf]

Supplementary Information

Nanotopological plate stimulates osteogenic differentiation through TAZ activation

Jun-Ha Hwang, Dong-Hyun Lee, Mi Ran Byun, A Rum Kim, Kyung Min Kim, Jung Il Park, Ho Taek Oh, Eun Sook Hwang, Kyu Back Lee, Jeong-Ho Hong

Supplementary Methods

Cell proliferation assay

MSC was seeded on flat or O70 nanopattern plate at a density of $1 \times 10^4/cm^2$. After cell adhesion to culture plate, cell viability was assessed by EZ-CYTOX cell viability assay kit (DoGEN, KR) according to manufacturer's instruction. In short, EZ-CYTOX reaction mixture was added to culture medium and incubated for 1 hr. After brief mixing, absorbance of reaction product was measured by microplate reader (Bio-rad, US) at 450nm wavelength. Cell viability was also analyzed at 2, 4, 6 days after seeding and data was plotted to make growth curve.

Supplementary Figures

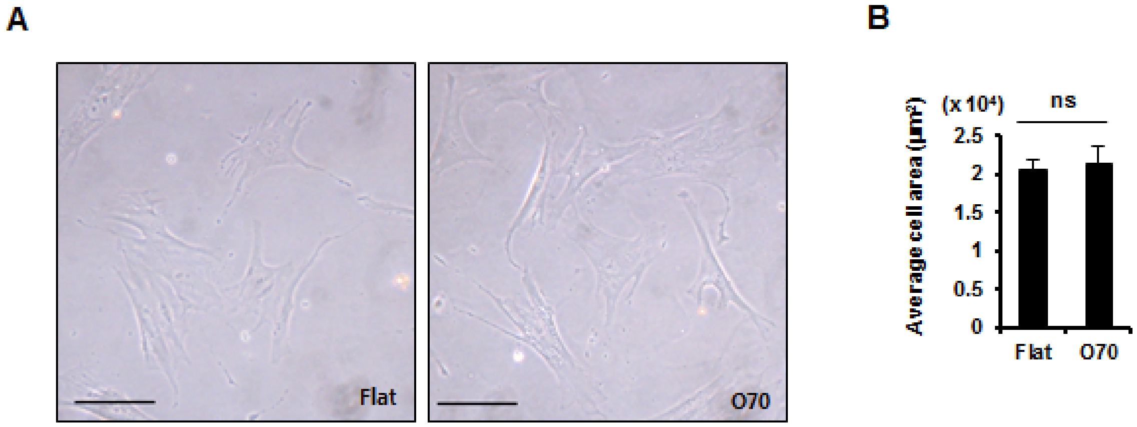

Supplementary Figure S1. There is no difference of cell area between flat and O70 surface. (A) MSC was seeded on flat or O70 nanopattern plate and bright field images were captured 24 hr after seeding. Scale bar = 200μm. (B) Cell spreading area was calculated by image J software from 6 images of each group. ns = not significant, t-test.

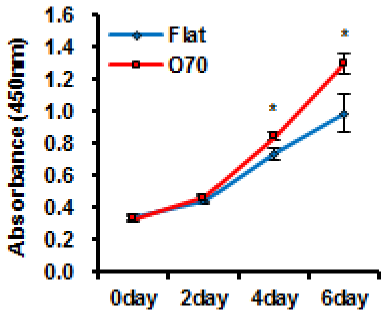

Supplementary Figure S2. Elevated MSC proliferation was observed on O70 nanopattern plate. MSC was seeded on flat or O70 nanopattern plate and cell viability was analyzed after cells adhere to culture plate. Cell viability assay was performed at 2, 4, 6 days after cell seeding and growth curve was plotted. n = 3 for each group, *$p < 0.05$, t-test.

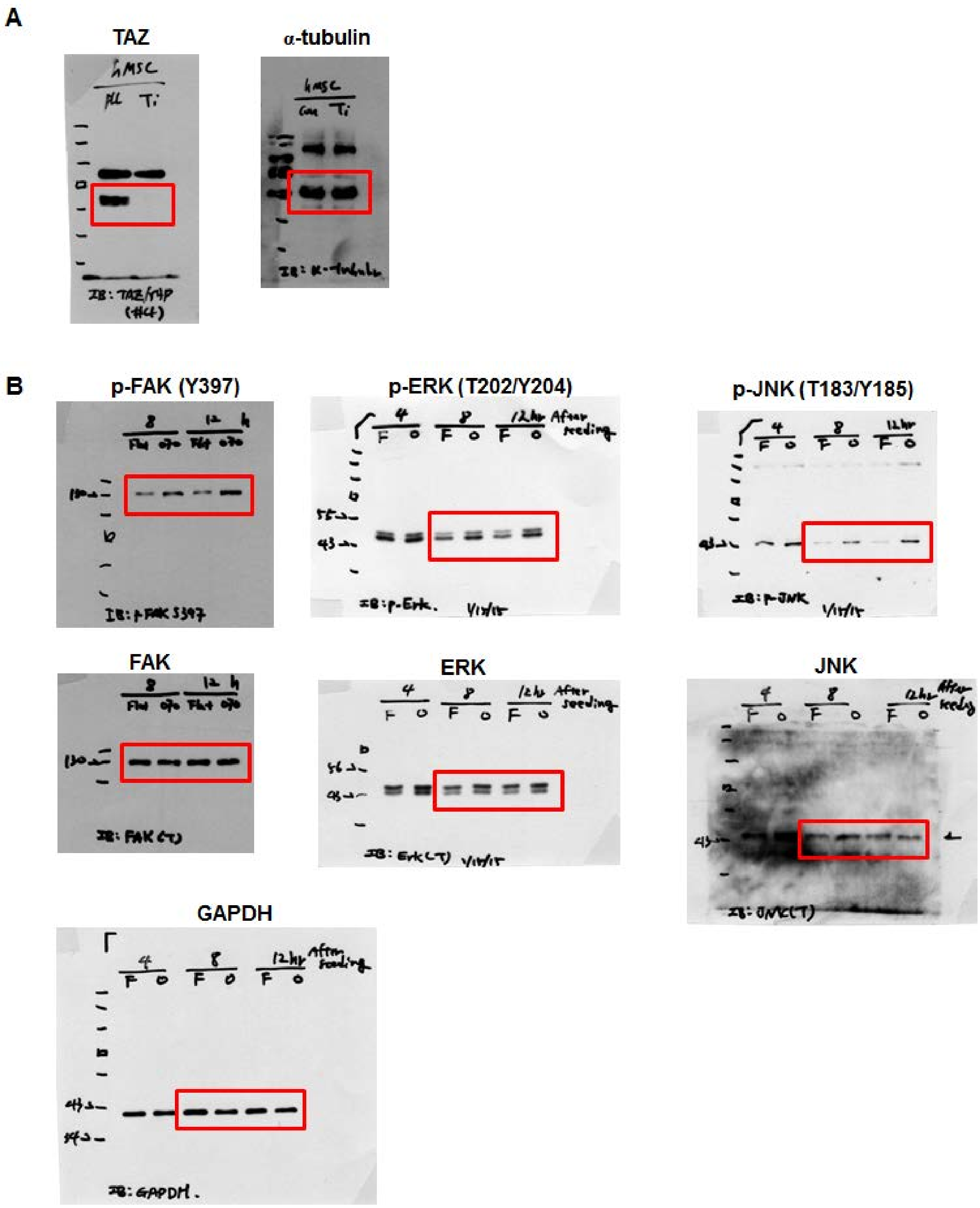

Supplementary Figure S3. Full length blots for Figure 4A (A) and Figure 6A (B)
